# Spectroscopic and Molecular Modeling Investigation on the Interaction between Folic Acid and Bovine Lactoferrin from Encapsulation Perspectives

**DOI:** 10.3390/foods9060744

**Published:** 2020-06-04

**Authors:** Iuliana Aprodu, Loredana Dumitrașcu, Gabriela Râpeanu, Gabriela-Elena Bahrim, Nicoleta Stănciuc

**Affiliations:** Faculty of Food Science and Engineering, Dunărea de Jos University of Galati, 800201 Galati, Romania; Iuliana.Aprodu@ugal.ro (I.A.); Loredana.Dumitrascu@ugal.ro (L.D.); Gabriela.Rapeanu@ugal.ro (G.R.); Gabriela.Bahrim@ugal.ro (G.-E.B.)

**Keywords:** folic acid, lactoferrin, interactions, molecular modeling, dietary, functional

## Abstract

The impact of thermal treatment on the ability of lactoferrin (FL) to bind folic acid (FA) was investigated by employing fluorescence spectroscopy, molecular dynamics and docking tests. The structural and conformational particularities of LF upon heating at 80 °C and 100 °C were first estimated based on the intrinsic fluorescence changes in respect to the native protein. The emission spectra indicated gradual unfolding events accompanied by Trp exposure with increasing temperature. In agreement with the experimental results, molecular modeling investigations showed that the secondary and tertiary structure of LF are slightly affected by the thermal treatment. Some minor unfolding events related particularly to the α-helical regions of LF were observed when the temperature increased to 100 °C. The LF fluorescence quenching upon FA addition indicated that a static mechanism stands behind LF-FA complex formation. Regardless of the simulated temperature, the hydrogen bonds played an important role in regulating the interaction between the protein and ligand. FA binding to LF equilibrated at different temperatures occurred spontaneously, and all complexes displayed good thermodynamic stability. The obtained results support the suitability of LF as biocompatible material, for obtaining micro- and nanoparticles for delivery of dietary supplements or for enhancing the functionality of target delivery systems.

## 1. Introduction

Nowadays, there is a great challenge in understanding the kinetics and thermodynamics of the interactions between small molecules and proteins, due to the ability of the later to bind different drugs and/or biologically active compounds as micro- and/or nano-carriers [1], with significant applications in clinical medicine, chemistry, food science and biotechnology.

Folates are a group of heterocyclic compounds with a 4-(pteridin-6-methylamino) benzoic acid skeleton, conjugated with one or more residues of l-glutamic acid [2]. The folates play key roles in fundamental biological processes including synthesis of nucleic acids and proteins, therefore it is highly important to assure the appropriate intake, thus preventing congenital diseases, such as neural tube defects [3]. Since mammalian cells are unable to synthesize folate, the exogenous supply of this vitamin is required for preventing nutritional deficiency [4]. Folic acid (4-[(pteridin-6-yl-methyl) amino] benzoic acid) (FA) is a synthetic and more stable form of folate, being usually used for fortification of different food matrices and for formulating different pharmaceuticals [2]. Several studies reported that FA is very sensitive to light, pH, temperature, oxygen exposure, ultraviolet light and heat, being degraded in compounds without biological activity [5,6]. In addition, FA is highly susceptible to degradation at acidic pH specific to the stomach, which also affects its bioavailability [7]. Therefore, this vitamin from group B requires designing delivery systems to protect, transport and control the release in the intestine, at neutral pH.

Bovine lactoferrin (LF) is an 80 kDa glycosylated protein with about 690 amino acid residues, out of which 13 are tryptophan (Trp) residues. The LF polypeptide chain is folded into two symmetrical lobes, which can reversibly and concomitantly binding to a ferric ion and a carbonate anion [8]. LF is an iron-binding protein belonging to the transferrin family [9], which is capable to inhibit bacterial growth [10]. Some other functions were attributed to LF, as described by da Silva et al. [11] and Padrão et al. [12], such as immunomodulatory activity, anti-inflammatory responses, and antimicrobial activity linked to the Fe-binding capacity, therefore allowing prevention of different diseases. For example, Gifford et al. [13] reported that LF contains at the N-terminus a small positively charged region termed lactoferricin (LFcin), which acts as antimicrobial peptide during inflammatory processes. Further, it has been demonstrated that LF forms a “shell” around activated neutrophils, thus inhibiting the release of NET fibers, the last step of NETosis [14].

Fluorescence spectroscopy is a suitable method for investigating the interaction between different ligands and biomacromolecules. The emission peaks, the efficiency of energy transfer, the lifetime of fluorophore, the values of fluorescence polarization, etc., provide valuable information on the structural changes and the fluctuations of the fluorophore surroundings in the macromolecules [15]. One of the major advantages of fluorescence spectroscopy is that the interaction process can be kinetically [association constant (*k_a_*)] and thermodynamically [binding constant (*K_b_*)] characterized. The temperature dependence of the kinetic parameters (*k_a_*) can be used to calculate the variations in Gibbs free energy (Δ*G‡*), and enthalpy (Δ*H‡*) and entropy (*T*Δ*S‡*) of activation, associated with a potential intermediate complex formation, which occurs during the interaction of free species or at dissociation of stable assemblies. In particular, the temperature dependent *K_b_* values allow the determination of the standard Gibbs free energy change (Δ*G^o^*), standard enthalpy change (Δ*H^o^*), and standard entropy change (Δ*S^o^*) of complex formation, thus allowing estimating the type of the forces involved in the interaction [8].

The fluorescence spectroscopy observations and the thermodynamics of protein interactions benefit from the single molecule level in silico observations, which facilitate deep understating of the most intimate mechanisms standing behind the macroscopic behaviour of the investigated biomolecules. In this respect, molecular dynamics simulations are a powerful tool for contributing to the elucidation of the biological macromolecules structure-function relationship, while understanding the physical basis of the atomic level events such as internal motions and further conformational changes. When dealing with the interaction of biomacromolecules with different compounds, a great contribution is provided by the molecular docking methods, which efficiently search for the most probable binging modes of the ligands on the receptors.

In the present study, the spectrofluorimetric measurements were combined with molecular docking and molecular dynamics simulation to investigate the interaction of FA with LF, such as to gather an in-depth understanding on the mechanism of molecular binding. The spectrofluorimetric titration data were exploited to extract data regarding the number of binding sites, binding parameters, and thermodynamics parameters for LF-FA complexation process. Further single molecule level details regarding the FA binding site on LF and the effect of complexation on the stability and particularities of the secondary structure elements of LF were collected by analyzing the LF-FA complexes obtained through molecular dynamics and molecular docking tests.

The results obtained in this study support the hypothesis of using food grade proteins as biocompatible and biodegradable materials, with high potential for obtaining micro- and nanoparticles for delivery of dietary supplements or for enhancing the functionality of target delivery systems.

## 2. Materials and Methods

### 2.1. Chemicals

LF from bovine milk, partially iron saturated (purity ≥ 85%, SDS-PAGE) and FA (purity > 97%) were purchased from Sigma (Sigma–Aldrich Co., St. Louis, MO, USA). LF was used without further purification. 2-Amino-2-(hydroxymethyl)-1,3-propanediol buffer (Tris base, 10 mM, pH 7.7) was used to keep constant pH value. All used salts for buffer preparation were of analytical grade and were dissolved in deionized water.

### 2.2. Preparation of Stock Solutions

Protein stock solution (1 mg/mL) was prepared by dissolving LF in 10 mM Tris base at pH 7.7. The protein concentration was determined spectrophotometrically using the extinction coefficient of 110.96 M^−1^ cm^−1^ at 280 nm [16]. FA stock solution (1 mg/mL) was made in 10 mM Tris base.

### 2.3. Heat Treatment

Plastic tubes (1 cm diameter) were filled with 0.15 mL of LF solutions containing 1 mg/mL protein solution in 10 mM Tris base buffer at pH 7.7. The samples were heated at 25, 80 and 100 °C for 15 min using a thermostatic water bath (Digibath-2 BAD 4, Raypa Trade, Barcelona, Spain). In order to avoid further thermal denaturation, the samples were then rapidly cooled by placing the tubes in ice water.

### 2.4. Fluorescence Spectroscopy

All fluorescence spectra were obtained from a LS-55 Luminescence Spectrometer (Perkin Elmer, Waltham, MA, USA), using a 1.0 cm quartz cell and a thermostat bath that keep temperature constant within ±0.1 °C. The width of slits for both the excitation and emission was set at 10.0 nm. The emission spectra were recorded at *λ_ex_* of 295 nm and *λ_em_* from 310 to 420 nm. The fluorescence intensity at the maximum emission wavelength (*λ_max_*) was used to calculate the binding constants.

In typical experiment, 0.1 mL of 1 mg/mL of LF solution was placed into the cuvette containing 3 mL of Tris base and was titrated by successive additions of 1 mg/mL of FA stock solution (increasing concentration from 0 to 11.3 × 10^−8^ M). The data obtained from fluorescence measurements were corrected for inner effect as described by Horincar et al. [16]. Each sample was scanned three times, the resulting spectra being represented as an average.

The fluorescence quenching may take place via static or dynamic mechanism. The fluorescence quenching mechanism was described by Stern-Volmer Equation (1), and the quenching constants of experimental data were obtained by regression analysis [17]:

(1) $$\frac{F_{o}}{F}=1+K_{SV}\times \left[Q\right]=1+k_{q}\times \tau_{o}\times \left[Q\right]$$

where *F_o_* and *F* are the fluorescence intensities in the absence and presence of the quencher, *K_SV_* is Stern-Volmer quenching constant determined by linear regression of the *F_o_/F* against [*Q*] plot, [*Q*] is the concentration of FA, *k_q_* is the fluorescence quenching rate constant, and *τ_o_* is the lifetime of the fluorophore without quencher and is equal to 10^−8^ s [18]. When static quenching takes place, the number of ligand binding sites, *n*, and apparent binding constant of protein-ligand complex, *K_a_*, can be estimated using the following equation [15]:

(2) $$ln\frac{\left(F_{o}-F\right)}{F}=lnK_{a}+n\cdot ln\left[Q\right]$$

### 2.5. Molecular Modeling Investigations

The three dimensional model of LF was taken from Brookhaven Protein Data Bank (PDB ID 1BLF; [19]). The protein model was refined, solvated, optimized and equilibrated at temperatures of 25 °C, 80 °C and 100 °C, using the sequence of steps detailed by Stănciuc et al. [20]. Molecular modeling simulations were carried out in parallelization conditions, using Gromacs (version 5.1.1.) software and Gromos96 43a1 force field [21].

The LF models equilibrated at different temperature were further used as receptors for FA binding in the docking procedure. PatchDock algorithm [22] based on the molecules shape complementarity was used to identify the most probable LF-FA complexes. The docking results were ranked considering the binding energy values, and for each simulated experimental condition the best solutions were analysed using PDBsum [23], PDBePISA [24] and LigPlot+ [25] tools, to collect significant details on the structure and interaction particularities of the LF-FA complexes.

### 2.6. Statistical Analysis

Statistical evaluation was performed using Minitab 19 (Minitab Inc., State College, PA, USA). Values are expressed as mean of triplicate values ± standard deviation. One-way ANOVA was used to identify differences between samples after passing two essential conditions: normality and homoscedasticity. Post-hoc analysis via Tukey method was employed at *p*-values lower than 0.05.

## 3. Results

### 3.1. Intrinsic Fluorescence Spectra

The intrinsic fluorescence of LF excited at 295 nm is mainly due to the Trp residues, and ligands binding, such as FA, might interfere with fluorescence emission [26]. Several studies indicated that measuring the intrinsic fluorescence quenching of LF, allows collecting valuable data on the binding characteristics and the change in the microenvironment of the fluorophore within the macromolecule [27,28].

**Figure 1:**
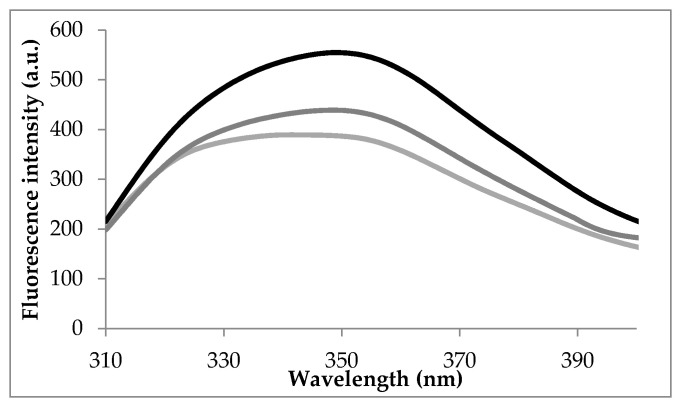
The fluorescence spectra of LF in the absence of the quencher.

To elucidate the thermodynamics of the interaction between FA and LF, the emission spectra of LF in the presence of various concentrations of FA were recorded upon excitation at 295 nm, after maintaining the protein for 15 min at 25 °C, 80 °C and 100 °C. Figure 1 displays the emission spectra of LF in the absence of the ligand.

At 25 °C, in the absence of the ligand, the emission spectra of LF show an emission maximum at 337 nm when excited at 295 nm. Heating at 80 °C and 100 °C caused significant red-shifts of 12 nm and 13 nm, respectively. These results suggested that heating resulted in conformational changes of the protein causing the movement of Trp residues towards a more polar microenvironment. In order to check this hypothesis, detailed analysis at single molecule level was carried out on LF equilibrated at different temperatures, in agreement with experimental conditions. Significant increase of the total surface available to the solvent (SAS) was noticed for Trp residues located in positions 8 (SAS increased from 3.90 Å^2^ at 25 °C to 11.16 Å^2^ at 80 °C and 39.95 Å^2^ at 100 °C), 361 (SAS increased from 1.72 Å^2^ at 25 °C to 9.69 Å^2^ at 80 °C and 10.32 Å^2^ at 100 °C) and 467 (SAS increased from 12.58 Å^2^ at 25 °C to 30.66 Å^2^ at 80 °C and 45.96 Å^2^ at 100 °C). Anyway, in addition to the differences in the exposure of the targeted fluorophore, the existence of the neighboring groups within interacting distances that may quench the fluorescence emission of Trp residues should be also factored.

The structural and conformational particularities of LF involve the presence of the single polypeptide chain folded into two symmetrical lobes (N and C lobes), linked through a hinge region containing parts of α-helix between amino acids 333 and 343 [29]. Bokkhim et al. [30] showed that the temperature increase from 25 to 80 °C caused the decrease of the α-helix content of native LF from 23.5% to 21.2%, accompanied by higher exposure of the hydrophobic residues. However, when considering the heat induced structural and conformational changes on LF, the iron-saturation degree should be considered. Ward et al. [31] suggested that LF may exists in different iron-saturated form, such as holo-LF, with iron saturation higher than 90%, native with iron saturation between 15% and 20% and iron-depleted, known as apo-LF. The denaturation temperature is highly dependent on the iron-saturation degree, with 91 °C for holo-LF [30], whereas native and apo-LF are more sensitive to heat, undergoing irreversible structural changes when heated at temperatures over 70 °C [32].

### 3.2. Quenching Experiments

**Figure 2:**
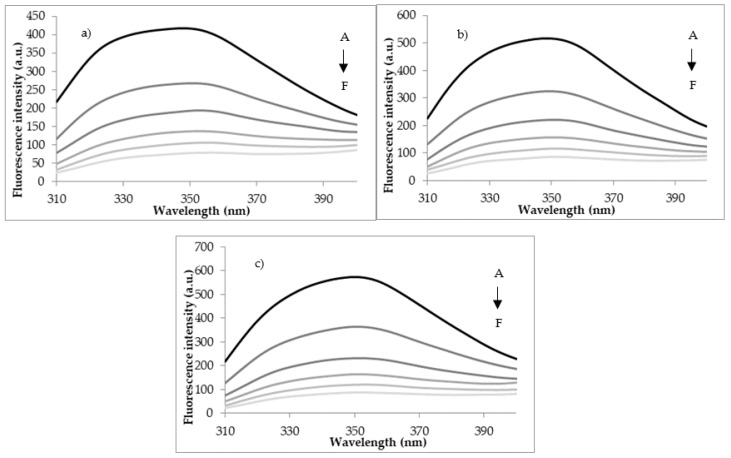
The fluorescence spectra of the interaction between LF thermally treated at 25 °C (**a**); 80 °C (**b**) and 100 °C (**c**) and FA. The FA concentration (from A–F) varied from 0 to 11.32 × 10^−8^ M.

The mechanism behind the fluorescence quenching experiments relies on the decrease of LF fluorescence intensity when the proteins-ligands inter-molecular interactions occur, involving collisional effect, ground-state complex formation, no-radical energy transfer, etc. The LF fluorescence quenching tests carried out by adding various concentrations of FA (ranging from 0 to 11.32 × 10^−8^ M) allowed assessing the accessibility of FA to the fluorophore groups of the protein. Increasing the concentration of FA up to 11.32 × 10^−8^ M resulted in the decrease of LF fluorescence, which was interpreted as FA binding to the protein (Figure 2). No significant differences in quenching extent as a function of temperature were observed, with a maximum value registered at 100 °C of approx. 85%.

The LF-FA docking models were carefully checked to identify any significant atomic level details which might help explaining the quenching mechanisms. No direct interaction between the ligand and the Trp residues from LF structure was observed in any of the investigated complexes. Anyway, the fluorescence quenching effect exerted by the folic acid when located in the vicinity of Trp residues should be considered. For instance, in case of the complex involving the protein equilibrated at 25 °C, the ligand is in direct contact with Ile^11^ which is located close neighborhood of Trp^8^. A close analysis of the complex involving the protein model equilibrated at 80 °C, indicated that that three of the amino acids in direct contact with the folic acid are located close to Trp^448^ (Leu^473^) and Trp^467^ (Ile^469^ and Gly^472^). Finally, in case of the protein heat treated at 100 °C, among the residues of the binding site Ile^129^ is placed in the close vicinity of the aromatic ring of Trp^125^.

**Figure 3:**
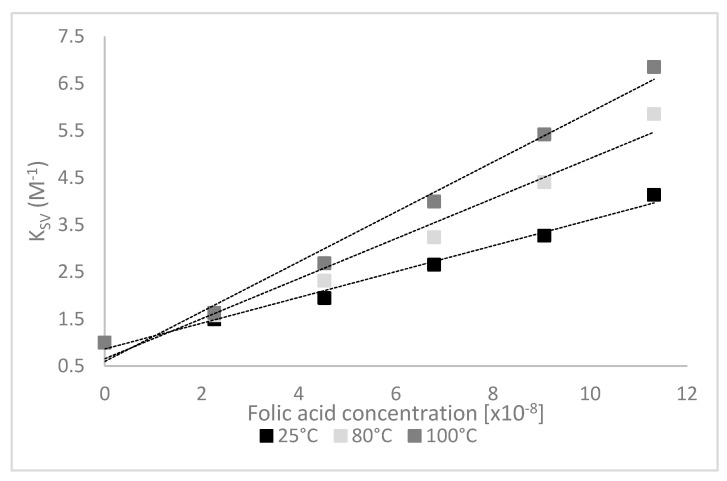
Stern-Volmer plots for the interaction of folic acid with heat-treated lactoferrin.

The fluorescence quenching mechanism is usually divided into static quenching and dynamic quenching. The dynamic quenching parameters of LF by FA, *K_SV_* and *K_a_* could be obtained from the experimental data using Stern-Volmer equation or modified Stern-Volmer equation. The Stern-Volmer plots are shown in Figure 3.

The emission quenching data were collected for complexes involving the protein heat treated at different temperatures, followed by applying the Stern-Volmer model such as to confirm the quenching mechanism that occurs in the LF-FA systems (Table 1).

As it can be seen from Table 1, the *K_SV_* values increased with temperature. The formation of protein-ligand complexes is usually characterized by the *K_SV_* decrease with increasing temperature [33]. However, it has been suggested that when complex formation is an endothermic process, *K_SV_* can increase with increasing temperature [34]. Additionally, the *k_q_* values were significantly higher than the maximum value of the diffusion-limited collisional quenching (2.0 × 10^10^ M^−1^ s^−1^) [15], indicating that the fluorescence quenching mechanism of LF is static, and the complexes formation was a potential entropy-driven process [35]. In our study, the results calculated by Stern–Volmer equation showed that the rate constant of the LF quenching procedure initiated by FA is much higher than the value of the scatter procedure (Table 1).

The Stern-Volmer relationship on a certain concentration condition suggested that a flexible fit effect may exist between FA and LF, as explained by Guo et al. [26]. For a static quenching binding mechanism, the fluorescence data were analyzed based on Equation (2), and values for the binding constant (*K_b_*) and the stoichiometry number (*n*) of the formed complexes were estimated (Table 1). From Table 1, it can be observed that, regardless of the temperature applied, there are no significant differences between the *K_b_* values, whereas the *n* values were calculated to be higher than 1, demonstrating the presence of more than one FA molecule bound on LF surface near the Trp residues. The corresponding *K_b_* values were about 1.68 ± 0.01 × 10^8^ M^−1^ at 25 °C and 1.66 ± 0.11 × 10^8^ M^−1^ at 100 °C.

Lower values were estimated by Guo et al. [26] for the binding interaction between tosufloxacin and bovine LF, who suggested values of 1.128 × 10^5^ M^−1^ for *K_SV_*, 1.128 × 10^13^ M^−1^ for *K_q_* and 7.849 × 10^4^ M^−1^ for *K_a_*, respectively.

Coelho et al. [8] studied the interaction of bovine LF with methylene blue and azure a dyes through surface plasmon resonance, fluorescence spectroscopy, and isothermal titration microcalorimetry. These authors estimated *K_SV_* values of 5.2 × 10^4^ M^−1^ at 25 °C for methylene blue and a higher affinity for azure A of 15.6 × 10^4^ M^−1^ at the same temperature. For the both dyes studied, these authors suggested an increase of the *K_sv_* values with increasing temperature, indicating a static quenching mechanism, with an entropy-driven process of the protein-ligand complex formation.

Four types of intermolecular forces are potentially involved in the LF-FA complex formation, namely hydrophobic interactions, van der Waals forces, electrostatic interactions, and hydrogen bonds [33]. The contribution of each type of intermolecular energy to the complex formation can be estimated based on the standard Gibbs free energy change (Δ*G^o^*), the standard enthalpy change (Δ*H^o^*), and the standard entropy change (Δ*S^o^*) of LF-FA interactions, as described by Coelho et al. [8].

The positive values of Δ*H^o^* and Δ*S^o^* showed in Table 2 indicate that the reaction between FA and LF is endothermic, suggesting that the main forces involved in the interactions are hydrophobic forces [35].

### 3.3. Molecular Modeling Investigation of FA Binding to LF

In silico calculations were further employed to simulate the thermal treatment of the protein at 25 °C, 80 °C and 100 °C and to estimate the impact on the FA binding properties. Unlike the experimental tests, the in silico approach allowed quantifying both reversible and irreversible structural changes of protein induced by thermal treatment. Temperature increase up to 100 °C caused gradual relaxation of the protein, accompanied by the 10% increase of the total LF surface. The increase of the solvation energy of folding (ΔG^f^) from −604.7 to −594.0 kcal/mol (Table 3) with the temperature indicates that the structural changes occurring in the solvated protein make it less thermally stable. Because of the molecular rearrangements within protein structure, the root mean square deviation (RMSD) obtained by least square fitting the FL models equilibrated at 25 °C and 100 °C was 10.27 Å. The temperature increase resulted in important changes of the secondary structure motifs. The most important changes were related to the helical content which dominate the secondary structure of LF. Indeed, the number of amino acids involved in α-helices decreased from 162 (corresponding to 23.5% α-helical content) at 25 °C, to 146 (corresponding to 21.2% α-helical content) at 80 °C and then to 107 (corresponding to 15.5% α-helical content) at 100 °C. At 25 °C and 80 °C the seven β-sheets among which three parallel, three antiparallel and one mixed were identified in the LF structure [20]. The thermal treatment at 100 °C affected one of the parallel β-sheet, because of the major heat induced changes in the dynamics of the hydrogen bonding involved in strands connection.

**Figure 4:**
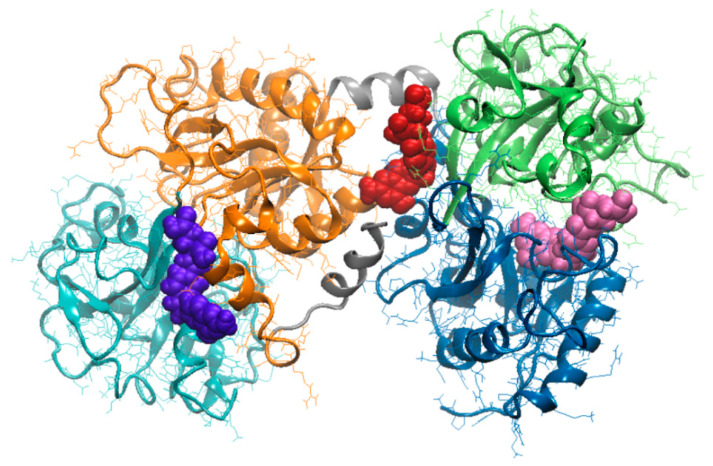
Superposition of the lactoferrin-folic acid complexes involving the protein pre-heated at different temperatures. The protein is represented in New Cartoon style. Domains N-1 (amino acids 1 to 90 and 251 to 333) and N-2 (amino acids 91 to 250) of the N lobe are represented in blue and lime, respectively. Domains C-1 (amino acids 345 to 431 and 593 to 676) and C-2 (amino acids 432 to 592) of the C lobe are represented in orange and cyan, respectively. The folic acid of the complexes involving proteins equilibrated at 25 °C, 80 °C and 100 °C, is represented in van de Waals style in mauve, violet and red, respectively.

The molecular docking tests indicated that the heat treatment affected the LF surface and internal cavities. Therefore, different FA binding sites were identified for the protein heated at different temperatures (Figure 4), and in none of the investigated complexes the ligand docking to the heat treated LF caused changes of the protein conformation.

As a consequence of the change of location of FA binding site, the interaction surface varied with the temperature in the 324.1–345.1 Å^2^ range (Table 3). When compared to the complex involving the native protein, a slight increase of the LF–FA binding energy was noticed at thermal treatment. However, it can be noted that the heat treated protein exhibited similar overall interaction forces toward FA, regardless of the temperature (Table 3).

In native state, the LF is able to accommodate the FA in a 30.53 Å deep cavity with volume of 1217.06 Å^3^, located in the core of the N lobe of the bovine protein. The FA binding site (Table 3) involves residues from both N-1 (amino acids located in positions 1 to 90 and 251 to 333) and N-2 (amino acids located in positions 91 to 250) domain of the N-terminal lobe of LF [20]. In fact, the FA binding site is located in a strongly protected hydrophobic environment (Figure 4), with no exposure to the aqueous environment, near the area where the iron is bound [36]. Given that FA penetrates into this cavity, strong attractive interaction involving dipoles buried into the nonpolar environment of the protein are noticed. In fact, two hydrogen bonds (Hb), which are a strong form of the dipole-dipole interaction, were found to connect the pteroyl group of FA by Thr^58^ (2.14 Å long Hb) and Arg^121^ (1.90 Å long Hb), therefore contributing to the stability of the complex formed between the native protein and the ligand.

The results of our simulations are in agreement with the observations of Nhan et al. [36], indicating higher thermal stability of the core regions of LF compared to the surface. It has been shown before that the environmental conditions influence the extent of domain closure of each lobe [20]. In particular, a general decrease of the relative distance between the amino acids of the iron binding site (Asp^60^, Tyr^92^, Tyr^192^ and His^253^) was observed at 80 °C. As expected, the ability of the LF to bind FA was modulated by the heat induced conformation changes, especially those involving the amino acids which define the opening of the cleft accommodating the iron ion. The docking results suggest that the protein equilibrated at 80 °C is able to bind the FA in a 9.97 Å deep cleft, with volume of 547.93 Å^3^, located on the surface of C lobe (Figure 4). The FA molecules is in contact with amino acids from both C-1 (amino acids located in positions 345 to 431 and 593 to 676) and C-2 (amino acids located in positions 432 to 592) [19] and is bridged through four Hb by Gly^472^ (2.14 Å long Hb), Lys^654^ (3.29 Å long Hb), Leu^661^ (3.31 Å long Hb), and Tyr^665^ (2.93 Å long Hb) residues.

In agreement with Stănciuc et al. [20], the LF heating to 80 °C resulted in slight compression (about 6%) of the hinge responsible for the connection of N- and C-lobes. Further temperature increase up to 100 °C resulted in a RMSD of 6.15 Å, calculated for the hinge relative to the native structure, causing the narrowing of the space between the two lobes of the LF, in such manner to suitable accommodate the FA molecules. The ligand established contacts mainly with the atoms of the N-lobe of the proteins, having the pteridine rings oriented toward Cys^405^ and Gly^406^ residues, belonging to the C1-lobe (Table 3), located in an area stabilised through a disulphide bond. As in the case of the other two complexes, the molecular interactions between the ligand and receptor was regulated by four hydrogen bonds with Arg^91^, Ile^129^, and Pro^232^. As in the case of the enzyme catalytic activity, Chen et al. [37] hypothesised that the H-bonds enhance the interactions between receptor and ligand when both donor and acceptor have equivalent H-bonding capabilities, stronger or weaker compared to the hydrogen and oxygen atoms of the surrounding water molecules. They proposed a hitherto donor-acceptor pairing mechanism, which minimizes competition with water, to explain the ability of H-bonds to regulate molecular interactions.

The LA binding to the LF occurs spontaneously, for all tested experimental conditions. The thermal treatment had no significant impact on the stability of the system. Although some unfolding events were noticed at increasing temperature, no impact on the rigid-body entropy change (TΔS^diss^) when dissociating the complex was observed (Table 3). The negative values of the solvation free energy gain upon assembly formation (ΔG^int^), including the effect of the satisfied hydrogen bonds between receptor and ligand, indicate the existence of hydrophobic interfaces within each analysed complex. Based on the lowest ΔG^int^ value (-2.36 kcal/mol), it can be stated that the highest affinity between the two compounds was obtained when pre-heating the protein. FA approached the cleft located on the C lobe surface of the LF pre-heated at 80 °C (Figure 4). In addition, when analysing the values of the free energy of assembly dissociation (ΔG^diss^), one can see that external driving forces are needed to dissociate the complex when dealing with LF treated at 80 °C (Table 3).

## 4. Conclusions

In this study, the fluorescence spectroscopy, molecular docking and molecular dynamics simulation were employed to investigate the interaction of folic acid with bovine lactoferrin. It was shown that the fluorescence of lactoferrin quenched due to the formation of the complex with folic acid through static mechanism, which was probably an entropy-driven process. The thermodynamic parameters gained after performing binding experiments involving the protein pretreated at various temperatures indicate the positive sign for both enthalpy and entropy changes and hence, hydrophobic interactions play important roles in the binding process.

The molecular modeling tests allowed identifying the small conformational rearrangements involving surface patches of side chains of the amino acids of the internal cavities impact ligand binding. Different folic acid binding sites were found through molecular docking in case lactoferrin pre-heated at different temperatures. When the lactoferrin model equilibrated at 25 °C and 80 °C was used as receptor, the amino acids involved in folic acid binding were located on N-lobe and C-lobe, respectively. When heating the protein at 100 °C, the best docking model revealed the folic acid exhibited high affinity toward a binding site located in the space between the N- and C-lobes.

These findings might help better understanding of the chemical mechanisms involved in the binding reaction between proteins and vitamins, and it is also looking forward for a further dietary supplement, in order to increase the bioavailability of the later. The results bring new evidence on the structure–function relationship of the complex, from the perspective of folic acid incorporation into innovative, protein-based functional matrices.

## Figures and Tables

**Table 1:** The binding parameters of the LF heat treated at different temperatures by FA.

T (°C)	*K_SV_* (10^8^ M^−1^)	*K_q_* (10^18^ mol^−1^s^−1^)	*K_b_* (10^8^ M^−1^)	*n*
25	0.28 ± 0.01 ^b^	0.28 ± 0.01 ^b^	1.68 ± 0.01 ^a^	1.24 ± 0.13 ^a^
80	0.48 ± 0.03 ^a^	0.48 ± 0.03 ^a^	1.67 ± 0.23 ^a^	1.30 ± 0.02 ^a^
100	0.52 ± 0.01 ^a^	0.52 ± 0.01 ^a^	1.66 ± 0.11 ^a^	1.32 ± 0.08 ^a^

Values are expressed as mean ± standard deviation. Superscript values that for the same column do not share the same letter (a, b) are significantly different at *p* < 0.001 based on Tukey method.

**Table 2:** The thermodynamic parameters for the association between heat-treated LF and FA.

T (K)	Δ*H^o^* (J·mol^−1^)	Δ*S*^o^ (J·mol^−1^K^−1^)	Δ*G*^o^ (J·mol^−1^)	*R ^a^*
298	15.29 ± 1.25 ^a^	0.47 ± 0.04	−124.77 ± 21.03	0.97
353			−150.62 ± 11.32	
373			−160.02 ± 16.98	

^a^–standard errors.

**Table 3:** Surface, energy and thermodynamic descriptors of the heat treated lactoferrin (LF) interacting with the folic acid (FA) molecule.

Descriptors	Temperature, °C		
	25	80	100
Surface descriptors			
Total protein surface, Å^2^	30,974.0	31,226.3	33,964.8
Total LF–FA interface area, Å^2^	324.1	341.1	345.1
Amino acids interacting with FA	Ile^11^, Ser^12^, Glu^15^, Ser^42^, Val^57^, Thr^58^, Leu^59^, Asp^60^, Arg^121^, Ser^185^, Phe^190^, His^253^, Gln^296^, Asp^297^, Leu^298^, Leu^299^	Ile^469^, Gly^472^, Leu^473^, Phe^475^, Asn^476^, Leu^589^, Ala^590^, Lys^654^, Leu^661^, Glu^664^, Tyr^665^, Thr^667^, Ser^668^, Asn^671^	Arg^91^, Ile^129^, Gly^130^, Thr^131^, Pro^232^, His^246^, Leu^247^, Ala^248^, Arg^249^, Leu^320^, Gly^321^, Asn^323^, Cys^405^, Gly^406^
Energy descriptors			
LF–FA binding energy, kcal/mol	−57.20	−46.03	−47.19
aVdW, kcal/mol	−24.97	−20.47	−22.45
rVdW, kcal/mol	5.59	4.24	4.86
Thermodynamic descriptors			
ΔG^f^, kcal/mol	−604.7	−602.9	−594.0
ΔG^int^, kcal/mol	−0.49	−2.36	−1.26
TΔS^diss^, kcal/mol	0.5	0.5	0.5
ΔG^diss^, kcal/mol	−0.9	0.1	−1.1

aVdW—attractive van der Waals energy; rVdW—repulsive van der Waals energy; ΔG^f^—solvation energy of folding; ΔG^int^—solvation free energy gain upon assembly formation; TΔS^diss^—rigid-body entropy change; ΔG^diss^—free energy of assembly dissociation.
